# Evaluation of Chronic Kidney Disease Progression in Dogs With Therapeutic Management of Risk Factors

**DOI:** 10.3389/fvets.2021.621084

**Published:** 2021-05-05

**Authors:** Sofía Perini-Perera, Javier Del-Ángel-Caraza, Alicia Pamela Pérez-Sánchez, Israel Alejandro Quijano-Hernández, Sergio Recillas-Morales

**Affiliations:** ^1^Facultad de Medicina Veterinaria y Zootecnia, Hospital Veterinario para Pequeñas Especies, Universidad Autónoma del Estado de Mexico, Toluca, Mexico; ^2^Facultad de Medicina Veterinaria y Zootecnia, Universidad Autónoma del Estado de Mexico, Toluca, Mexico

**Keywords:** chronic kidney disease, progression disease, risk factors, IRIS stages, dogs

## Abstract

This research was performed to describe the characteristics of the progression of naturally occurring chronic kidney disease (CKD) in dogs, together with the management of identified risk factors, following the International Renal Interest Society recommendations. Dogs diagnosed and staged with CKD, and with a longitudinal follow-up from the moment of diagnosis of up to a maximum of 730 days, were included. A total of 545 dogs that presented risk factors for the development of CKD were analyzed, out of which 36 met the inclusion criteria. Advanced age was identified in 80.6% of cases. Initiation risk factors were represented by inflammatory/infectious diseases, history of anesthetic-surgical procedures, heart disease, neoplasms, endocrinopathies, and exposure to nephrotoxic drugs. During the follow-up period, progression of CKD was identified in 47.2% of the cases, being more salient in advanced stages. Serum symmetric dimethyl arginine (SDMA) was the only glomerular filtration rate (GFR) marker which displayed differences among studied times during early stages of CKD, associated with the disease progression and decline of renal function. A significant difference between the survival curves in early and advanced CKD stages was observed. The factors related to decreased survival were hyperphosphatemia, anemia, and low body condition score (BCS). No differences were found between the presence of arterial hypertension and renal proteinuria and decreased survival. Furthermore, CKD diagnosis based on the persistent finding of abnormalities in early disease markers, such as serum symmetric dimethyl arginine increase and/or renal proteinuria, and timely therapeutic management of risk factors, allowed for CKD stabilization, reducing progression to advanced stages, and favoring higher survival rates.

## Introduction

Chronic kidney disease (CKD) is considered a frequent pathology in everyday small animal practice and tends to be progressive and irreversible once parenchymal mass reduction reaches a critical limit (1, 2). It can be caused by a wide variety of pathologies and events or factors, and its severity can be determined through different clinical findings (1). In various geographical regions of Latin America, there is insufficient access to renal replacement therapies in veterinary medicine. It is also known that late CKD diagnosis is associated with poor prognosis. These facts account for enough reasons to focus all efforts on early diagnosis, favoring, along with therapeutic management implementation, the stabilization of the disease, reducing the speed of its progression, and improving the quality and survival time of patients (2, 3).

To achieve CKD diagnosis during early stages, research must be done in order to find related risk factors **(**RF), using information from the patient's clinical history along with concurrent disease diagnosis, since characteristic clinical signs are not common in early CKD stages (4). This diagnostic approach has been created by the International Renal Interest Society (IRIS) (5) and proposes that once patients exposed to RF are identified, a series of tests should be made in order to acknowledge the presence of kidney disease (4). Finding a maintained increase in glomerular filtration rate (GFR) markers serum concentration is compatible with the disease. Routine evaluation of GFR consists of serum creatinine concentration (sCr) determination. However, it has considerable limitations. As a result of this, novel GFR markers are being studied, such as symmetric dimethyl arginine (SDMA), which appears to be an endogenous kidney biomarker suitable as a screening test for detection of CKD when performed in conjunction with sCr determination, nevertheless, much remains to be learned about its qualities (6). After diagnosis, staging and substaging the disease enables the establishment of a more accurate prognosis and guides decision making regarding therapeutic management (4, 7). Through different tests, it is possible to identify progression RF, such as renal proteinuria and systemic hypertension, which are related to a faster rate of disease progression (8), and end-stage RF, such as hyperphosphatemia, anemia, and loss of body condition score (BCS), which are associated to increased morbidity and mortality (9–11).

Although there are numerous evidences concerning the benefit of therapeutic management of RF (8), there are still inconsistencies regarding the true risk these factors represent as well as the optimal moment for therapeutic management implementation (12–14), as a consequence of the lack of prospective studies that broadly evaluate the results of therapeutic interventions in dogs with CKD (6, 11, 15).

The objective of this work was therefore to recognize the characteristics of naturally occurring CKD progression in dogs, with the management of identified RF following IRIS recommendations.

## Materials and Methods

### Dogs and Ethics Statement

Dogs that attended the Hospital Veterinario para Pequeñas Especies of the Veterinary Faculty -Universidad Autónoma del Estado de Mexico, Toluca-Mexico, for clinical consultation, were evaluated between January 2016 and January 2019. In this study, only dogs with CKD diagnosis and staging, with at least two re-evaluations and a longitudinal follow-up from the moment of diagnosis of up to a maximum of 730 days, were included (180 days for dogs in stages I or II and 60 days for dogs in stages III and IV). Due to CKD severity of some dogs in stages III and IV, no further evaluations were accomplished; they were excluded from following evaluations. Staging is explained in Section Diagnosis, Staging, and Longitudinal CKD Monitoring.

Medical management was carried out to diagnose CKD and concomitant pathologies, as well as to therapeutically manage factors involved in the aggravation of this disease, according to IRIS recommendations for that matter. No experimental interventions nor additional management generating pain or stress was performed to the studied cases, all procedures were performed following the internationally established high standards of good practice and animal welfare (16), informed consent of owners was obtained for the performance of necessary diagnostic procedures in their dogs, and the Institutional Committee for the Care and Use of Laboratory Animals of the own institution considered that all the procedures performed on the animals studied were only diagnostic and/or therapeutic in relation to their disease, not performing any intervention or experimentation procedure.

### Study Design

Dogs studied were identified with RF for CKD, determined from clinical history and general physical examination (17, 18). Initially, systemic blood pressure was measured non-invasively by the oscillometric method (Vet20, SunTech Medical, NC, USA), following the protocol described by Acierno et al. (19). After an 8-h fasting period, blood samples were collected by jugular venipuncture to perform a complete blood count and an integral biochemical profile (ProCyte Cx and Catalyst One, IDEXX Laboratories, Maine, USA) with SDMA determination by liquid chromatography-mass spectroscopy (IDEXX Laboratories, Maine, USA). Urine samples were obtained by cystocentesis, and a complete urinalysis was performed, determining urinary-specific gravity (USG) with a manual refractometer. Urine samples without active sediment were used to determine the magnitude of proteinuria by the urine protein:creatinine ratio (UPC) through spectrophotometry. Urinary protein was determined by the pyrogallol red and molybdate in acidic medium reaction and urinary creatinine by Jaffe's modified reaction (BTS-350 spectrophotometer, Protein-COD 11501; Creatinine-COD 11502 BioSystems, Barcelona, Spain).

#### Diagnosis, Staging, and Longitudinal CKD Monitoring

Evidence of a reduction in GFR with a USG <1.030 was considered suggestive of renal disease according to IRIS criteria (20) [SDMA >14 μg/dl and/or sCr >124 μmol/L]. The reassessment of renal function was performed within 30–60 days to confirm the problem's chronicity and stability (21). Whenever renal proteinuria was detected, its reassessment was performed at a 15- to 21-day interval to confirm its persistency (22). The disease staging was performed based on IRIS proposed criteria in 2017 (20).

The therapeutic management of RF started after CKD staging and substaging (20, 22). Patients in stage I of the disease with persistent proteinuria were fed a renal therapeutic diet,^1^ and an ACEI or ARA was incorporated to the medical management (13, 22–24). Patients in azotemic stages received a renal therapeutic diet and a phosphate binder with food every 12 h^2^. Moreover, persistent renal proteinuria in these patients was managed as described above. Patients in advanced stages (III and IV) of the disease were supplemented with iron-dextran when hematocrit diminution or anemia was detected. In all cases, necessary procedures were carried out to achieve control of signs associated with uremia and its complications (antiemetics, antisecretory drugs, etc.) (8, 20), and the specific medical management for the concurrent diseases present in each clinical case.

Patients' follow-up was carried out through periodic reevaluations, performing the necessary therapeutic adjustments in all cases (8). It is worth mentioning that staging and therapeutic management criteria and recommendations were based on IRIS publications in 2016–2017 (5, 20), since these were the valid criteria available during the study period. Nevertheless, data analysis was performed using the more current staging criteria (7).

#### Study Times, Study Groups, and Progression Criteria

The reevaluation at which CKD diagnosis was confirmed, staging properly performed, and RF management set up, was defined as the initial time (*T*_0_). The final time (*T*_F_) corresponded to the end of the follow-up period for each case. The laboratory evaluations used in progressions analysis were carried out on average at day 180 (days 60–210), day 365 (days 335–395), day 540 (days 510–570), and day 730 (days 700–760).

Patients were assigned into four groups according to the IRIS 2019 criteria for CKD staging (7). Group 1 (G_1_) included patients with persistent renal proteinuria (UPC >0.2) or with a slight GFR decrease (SDMA 15–17 μg/dl). Group 2 (G_2_) included patients with mild azotemia (sCr 125–250 μmol/L; SDMA 18–35 μg/dl). Group 3 (G_3_) included patients with evidence of moderate azotemia (sCr 251–440 μmol/L; SDMA 36–54 μg/dl), and group 4 (G_4_) included patients with severe azotemia (sCr >440 μmol/L; SDMA ≥54 μg/dl). For data analysis, patients included in G_1_ and G_2_ were considered to be in early CDK stages, and patients included in G_3_ and G_4_ in advanced CKD stages. Increase in CKD severity in different stages of the disease or death associated with uremic syndrome was considered a progression.

#### Risk Factors, Progression, and Mortality Analysis

The CKD RF were analyzed (17, 18) considering age a susceptibility RF and concurrent diseases, history of anesthetic/surgical procedures, and exposure to nephrotoxic drugs as initiation RF. Renal proteinuria and systemic hypertension as progression RF, while hyperphosphatemia, anemia, hypoproteinemia, and cachexia were considered end-stage RF. Progression and mortality proportions and survival rates of the population were also analyzed.

#### Statistical Analysis

Statistical analysis was performed using the software package Graphpad® Prism 8.1.1 (www.graphpad.com/scientific-software/prism/ California, USA), considering interest variables age, sex, breed, SDMA, sCr, serum urea, serum phosphorus, serum calcium, serum albumin, USG, UPC, hematocrit, systolic blood pressure (SBP), and BCS. The presence of a *p* < 0.05 was considered statistically significant in all cases. Normality was evaluated by the Shapiro-Wilk test, and since no normality was detected, variables were presented as medians and intervals (0.025–0.975) and non-parametric tests were used. Differences in interest variables among study groups were determined using the Kruskal-Wallis test (Dunn's *post hoc*). Moreover, *T*_0_ and *T*_F_ in early and advanced CKD stages were compared by the Wilcoxon test, and the association between variables was assessed by *X*^2^ test. For survival analysis, survival median and range (0.025–0.975) were determined. The Kaplan-Meier test was performed for each study group and interest variable, categorizing the last ones based on their cut-off points, considering in each case the established reference interval. The presence of significant differences among survival curves was analyzed by the log-rank test (Mantel-Cox), and the corresponding hazard ratios were also recorded.

## Results

In this study, 545 dogs that presented CKD RF were evaluated. In 162 of these cases, a persistent GFR decrease and/or renal proteinuria was identified; however, out of these, only 36 cases met all inclusion criteria. In the 36 studied cases, the median age at *T*_0_ was 10 years with an interval of 3–15 years, detecting the presence of age as a susceptibility RF in 80.6% (29/36) of the cases. Of the studied dogs, 61.1% (22/36) were male, and the rest were female. The breeds identified more frequently were miniature poodle (*n* = 10), chihuahua (*n* = 4), miniature schnauzer (*n* = 2), standard dachshund (*n* = 2), boxer (*n* = 2), and cross-breed (*n* = 2). Other breeds were represented by a single specimen (*n* = 14). Based on breed size, 66.7% (24/36) of the cases were classified as small, 13.9% (5/36) as medium, 16.7% (6/36) as large, and 2.78% (1/36) as giant.

The disease's initiation RF identified were inflammatory/infectious diseases, history of anesthetic/surgical procedures, heart disease, neoplasms, endocrinopathies, and exposure to nephrotoxic drugs. Periodontal disease was identified in 94.4% (34/36) of the cases, history of at least one surgical procedure in 69.4% (26/36), osteoarthritis in 38.9% (14/36), degenerative valvular disease in 30.6% (11/36), neoplasms, such as carcinomas and sarcomas, in 30.6% (11/36), infectious diseases, such as chronic leptospirosis and ehrlichiosis, in 19.4% (7/36), endocrine diseases, such as hyperadrenocorticism, hypothyroidism, and diabetes mellitus, in 16.7% (6/36), pyometra in 13.9% (5/36), chronic dermatitis in 13.9% (5/36), exposure to nephrotoxic drugs in 13.9% (5/36), and renal amyloidosis in one case.

Chronic kidney disease progression was identified in 47.2% (17/36) of the cases, being more evident in advanced stages [*p* = 0.0155]. In cases that progressed to more advanced stages, the concurrent diseases identified were heart disease in 54.5% (6/11), neoplasms in 18.2% (2/11), severe chronic proteinuria in 9.1% (1/11), chronic leptospirosis in 9.1% (1/11), and renal amyloidosis in 9.1% (1/11). It should be noted that in patients in G_3_ and G_4_ that died due to CKD severity, active concurrent diseases were not identified.

**Figure 1:**
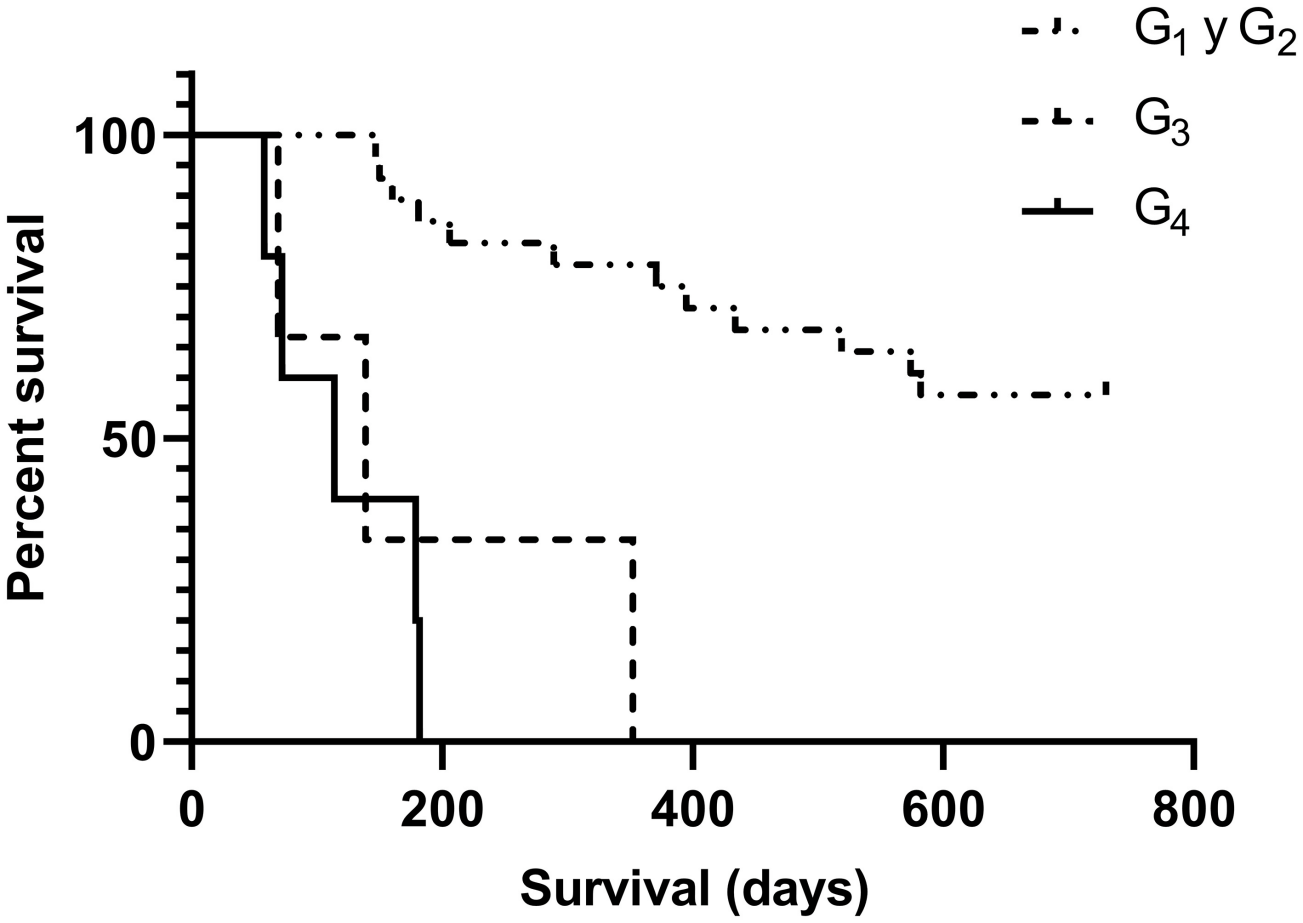
Survival analysis at different stages of CKD determined by the Kaplan-Meier test. A highly significant difference was observed between the survival curves of patients in early stages -G_1_ and G_2_-, G_3_, and G_4_ (*p* < 0.0001).

The overall population survival was 44.4% (16/36), being mortality attributed in these cases to different concomitant pathologies and complications derived from CKD's severity, mainly in advanced CDK stages. Specific mortality associated with CKD was 19.4% (7/36), and proportional mortality was 35.0% (7/20). When comparing survival time between early and advanced CKD stages, a significant difference was observed between the survival curves [*p* < 0.0001] (Figure 1). Median survival in early CKD stages was 730 days, with an interval of 150–730 days, and 127 days, with an interval of 60–322 days, in advanced CKD stages.

Based on laboratory test results at *T*_0_, 77.8% (28/36) of the cases were classified in early CKD stages and 22.2% (8/36) in advanced CKD stages. The 55.6% (20/36) of the studied dogs were classified in G_1_, and during the follow-up period, 35.0% (7/20) evidenced progression to G_2_, of which 85.7% (6/7) progressed by day 180 and 14.3% (1/7) by day 360. Furthermore, 55% (11/20) of the cases in this group survived until the last evaluation on day 730. The overall mortality rate of G_1_ was 45.0% (9/20), and the median survival time was 355 days. Causes of death in this group were associated with the different concurrent diseases present at that time, impeding linking CKD as death cause in any of these cases. G_2_ included 22.2% (8/36) of the cases studied, and during the follow-up period, 37.5% (3/8) evidenced progression to G_3_, of which 66.7% (2/3) progressed at day 180 and 33.3% at day 360. Furthermore, 62.5% (5/8) of the cases in this group survived until the last evaluation at day 730. The overall mortality of this group was 37.5% (3/8), and the median survival time of the deceased was 289 days. Deaths were associated to concurrent diseases present in all cases. The 8.3% (3/36) of the dogs was classified in G_3_, and CKD progression was observed in 66.7% (2/3) of the cases. The median survival period of this group was 139 days, with an interval of 73–341 days. Overall mortality in G_3_ was 100%, with a specific mortality of 66.7% (2/3). Of all the cases, 13.9% (5/36) were classified in G_4_, all of which manifested disease progression (death associated with uremic syndrome); therefore, specific mortality was 100% (5/5). The median survival time was 114 days, with a range of 60–182 days.

To analyze progression and end-stage RF, the behavior of the different variables in each study groups at *T*_0_ was studied (Table 1). A significant difference was observed in GFR markers (SDMA and sCr) between G_1_ vs. G_2_, G_3_, and G_4_ [*p* < 0.0001]. Concurrently, serum urea showed a significant difference between G_1_ and G_4_ [*p* = 0.0003]. For serum phosphorus, differences were noted between G_1_ and G_4_ and between G_2_ and G_4_ [*p* = 0.0037], observing a tendency toward its increase as CKD severity worsened. It is worth mentioning that cases with overt hyperphosphatemia were observed in all groups, being this event more frequent in G_3_ and G_4_. The USG showed differences between G_1_ and G_4_ [*p* = 0.0090] and a tendency toward its decrease as CKD severity increased. Moreover, 75.0% (15/20) of the cases in G_1_ presented only renal proteinuria as inclusion criteria, out of which 46.7% (7/15) showed marked hypersthenuria (USG >1.030). The hematocrit showed significant differences between G_1_ and G_4_ and between G_2_ and G_4_ [*p* = 0.0016], with a tendency toward its decrease as CKD severity increased. Hematocrit medians in G_3_ and G_4_ were below the reference interval (0.37–0.55 L/L). The BCS also showed a significant difference among groups [*p* = 0.0378], tending to decrease as the severity of the CKD increased.

**Table 1:** Variables of interest related to progression and end-stage risk factors (RF), represented for each study group at the time of diagnosis (*T*_0_), and the comparison between these by the Kruskal-Wallis test.

**Variables [reference interval]**	**G_**1**_**	**G_**2**_**	**G_**3**_**	**G_**4**_**	***P*-value**
Number of dogs	20	8	3	5	
SDMA [≤14 μg/dl]	12.0 (6.48–17.00)a	20.5 (14.40–33.40)b	40.0 (39.10–47.60)b	56.0 (34.70–86.40)b	<0.0001^*^
sCr [≤124 μmol/L]	81.5 (39.28–124.00)a	153.2 (107.23–236.16)b	320.0 (225.95–394.10)b	610.0 (446.70–715.22)b	<0.0001^*^
Urea [2.5–9.6 mmol/L]	5.80 (2.11–16.96)a	9.45 (5.54–16.19)ab	23.20 (17.12–29.28)ab	43.12 (38.45–46.24)b	0.0003^*^
sPhos [0.9–1.9 mmol/L]^c^	1.39 (0.72–1.96)a	1.26 (0.84–1.96)a	1.71 (1.62–3.13)ab	2.64 (1.66–4.64)b	0.0037^*^
sCa [1.98–3.0 mmol/L]	2.55 (2.17–3.02)	2.56 (2.24–2.89)	2.53 (2.48–2.88)	2.43 (2.12–2.77)	0.8708
sAlb [22–39 g/L]	28.0 (19.85–33.05)	28.1 (24.35–32.65)	25.6 (22.18–25.98)	28.0 (21.20–29.90)	0.2476
USG [>1.030]	1.026 (1.004–1.047)a	1.017 (1.011–1.036)ab	1.009 (1.008–1.013)ab	1.012 (1.007–1.014)b	0.0090^*^
UPC [<0.2]	0.91 (0.22–6.98)	1.50 (0.27–2.40)	1.05 (0.48–2.04)	0.64 (0.32–1.68)	0.8175
Hematocrit [0.37–0.55 L/L]	0.49 (0.39–0.64)a	0.50 (0.37–0.61)a	0.36 (0.34–0.48)ab	0.23 (0.12–0.43)b	0.0052^*^
SBP [<140 mmHg]	140.5 (105.7–166.0)	144.5 (133.2–156.0)	147.0 (138.5–238.2)	121.5 (106.7–128.9)	0.0563
BCS [5-6]	5 (3.0–7.0)	5 (3.2–7.7)	3 (3.0–4.0)	3 (1.1–6.6)	0.0378^*^

*BCS, body condition score; SBP, systolic blood pressure; sCr, serum creatinine; SDMA, symmetric dimethylarginine; sPhos, serum phosphorus; sCa, serum calcium; sAlb, serum albumin; UPC, urine protein:creatinine ratio; USG urinary specific gravity*.

*Variables are expressed as medians and intervals (0.025–0.975)*.

c *Intervals for serum phosphorus were in accordance to the CKD stage based on IRIS 2019 criteria*.

* *p < 0.05, statistically significant differences based on the Kruskal-Wallis test*.

*The presence of different literals (a, b) indicates statistically significant differences between the groups in Dunn's post hoc analysis (p < 0.05)*.

While searching for differences among *T*_0_ and *T*_F_ regarding interest variables, a statistically significant difference was observed for cases in early CKD stages concerning SDMA's concentration [*p* = 0.0158], showing with a tendency toward its increase as CKD severity increased. Moreover, the hematocrit showed a significant decrease among study times [*p* = 0.0093], evidencing a slight variation on its medians while being at both times within the reference interval. Other progression and end-stage RF analyzed, such as renal proteinuria, systemic hypertension, hyperphosphatemia, hypoalbuminemia and low BCS, did not show significant differences among the referred study times. It is noteworthy that serum phosphorus, albumin, and BCS medians remained within the reference interval in these cases. For patients in advanced CKD stages, when comparing variables at *T*_0_ and *T*_F_, a statistically significant difference was observed for the hematocrit [*p* = 0.0391], evidencing a significant decrease over time and anemia-compatible medians at both times. The values of SDMA, sCr, serum urea, phosphorus, albumin, UPC, SBP, and BCS showed no significant differences between study times (Table 2).

**Table 2:** Comparison between interest variables at initial time (*T*_0_) and final time (*T*_F_) for patients with early CKD stages -G_1_ and G_2_- and advanced CKD stages -G_3_ and G_4_-, by Wilcoxon test.

**Variables [Reference interval]**	***T*_**0**_**	***T*_**F**_**	***P*-value**
**Early CKD stages (*****n*** **=** **28)**			
SDMA [≤14 μg/dl]	13.5 (6.68–28.93)	15.0 (6.03–46.18)	0.0158^*^
sCr [≤124 μmol/L]	99.5 (41.08–202.32)	100.5 (50.10–402.20)	0.0539
Urea [2.5–9.6 mmol/L]	7.15 (2.41–16.90)	7.90 (1.90–35.00)	0.1413
sPhos [0.9–1.5 mmol/L]^c^	1.39 (0.73–2.06)	1.38 (0.69–3.38)	0.6788
sCa [1.98–3.0 mmol/L]	2.55 (2.18–3.02)	2.58 (2.16–3.03)	0.7951
sAlb [22–39 g/L]	28.0 (21.05–33.33)	28.5 (17.50–34.90)	0.3854
USG [>1.030]	1.021 (1.005–1.046)	1.018 (1.004–1.038)	0.1438
UPC [<0.2]	1.17 (0.23–6.64)	0.49 (0.10–6.02)	0.2204
Hematocrit [0.37–0.55 L/L]	0.49 (0.37–0.63)	0.48 (0.30–0.61)	0.0093^*^
SBP [<140 mmHg]	144.0 (107.5–164.6)	135.0 (108.0–177.0)	0.3086
BCS [5-6]	5.0 (3.0–7.3)	4.5 (2.4–7.0)	0.1252
**Advanced CKD stages (*****n*** **=** **8)**			
SDMA [≤14 μg/dl]	44.5 (34.88–85.20)	51.5 (33.05–70.00)	0.9219
sCr [≤124 μmol/L]	489.5 (238.33–709.38)	394.0 (265.53–1070.07)	0.9453
Urea [2.5–9.6 mmol/L]	38.20 (17.76–46.40)	38.10 (20.80–71.63)	0.8750
sPhos [0.9–1.9 mmol/L]^c^	2.45 (1.60–4.57)	1.67 (1.28–5.88)	>0.9999
sCa [1.98–3.0 mmol/L]	2.51(2.14–2.88)	2.57 (1.51–3.04)	0.9375
sAlb [22–39 g/L]	25.8 (21.18–29.83)	26.0 (20.63–27.86)	0.7500
USG [>1.030]	1.011 (1.007–1.014)	1.012 (1.009–1.018)	0.2344
UPC [<0.2]	0.85 (0.33–2.02)	1.15 (0.37–3.51)	0.9688
Hematocrit [0.37–0.55 L/L]	0.32 (0.13–0.48)	0.26 (0.14–0.44)	0.0391^*^
SBP [<140 mmHg]	129.0 (107.4–228.6)	128.0 (92.2–157.5)	0.0781
BCS [5-6]	3 (1.2–6.5)	3 (1.0–6.5)	0.9999

*BCS, body condition score; SBP, systolic blood pressure; sCr, serum creatinine; SDMA, symmetric dimethylarginine; sPhos, serum phosphorus; sCa, serum calcium; sAlb, serum albumin; UPC, urine protein:creatinine ratio; USG, urinary specific gravity*.

*Variables are expressed as medians and intervals (0.025–0.975)*.

c *Intervals for serum phosphorus were in accordance to the CKD stage based on IRIS 2019 criteria*.

* *p < 0.05, statistically significant differences based on the Wilcoxon test*.

**Figure 2:**
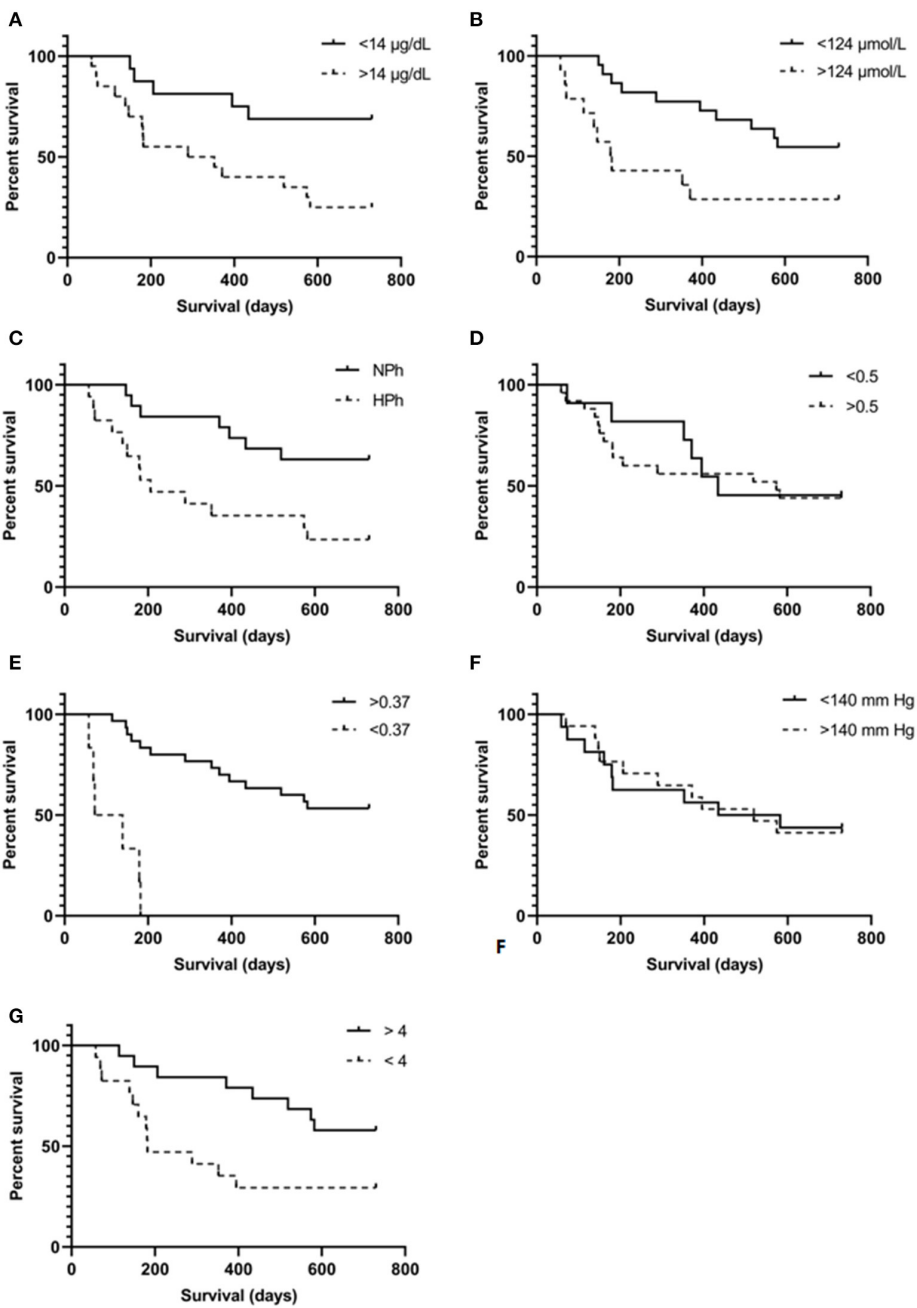
Kaplan-Meier survival curves were determined for each risk factor associated interest variable, using specific cut-off point for each. **(A)** Increased symmetric dimethylarginine, cut-off point 14 μg/dl; **(B)** increased serum creatinine, cut-off point 124 μmol/L; **(C)** hyperphosphatemia, cut-off point for serum phosphorus concentration was 1.5 mmol/L for G_1_ and G_2_, 1.6 mmol/L for G_3_, and 1.9 mmol/L for G_4_ (all values above the upper value of the interval were considered compatible with hyperphosphatemia); **(D)** urine protein:creatinine ratio, cut-off point 0.5; **(E)** hematocrit, cut-off point 0.37 L/L; **(F)** systolic blood pressure, cut-off point 140 mm Hg; **(G)** body condition score, cut-off point 4.

When analyzing the relationship between the presence of progression and end-stage RF identified at *T*_0_ and survival time, we noticed that an increase in serum SDMA concentration, hyperphosphatemia, anemia, and low BCS-conditioned survival (Figure 2). It was observed that patients with serum SDMA levels above the reference interval had 3.5 times higher death risk than those with SDMA below the cutting point. However, increased sCr levels were not statistically related to survival in these patients [*p* = 0.0211]. Cases in which hyperphosphatemia was noticed had 3.3 times higher death risk compared with normophosphatemic dogs. As for anemic patients, they had 8.5 times higher death risk than non-anemic patients. Additionally, patients with low BCS had 2.7 times higher death risk than those who had normal BCS. It is worth mentioning that in the studied population, no statistically significant difference was observed among survival curves for RF such as systemic hypertension and renal proteinuria (Table 3).

**Table 3:** Hazard ratios for analyzed variables classified based on cut-off values determined at *T*_0_.

**Variables**	**Reference cut-off**	***P*-value**	**Hazard ratio**	**95% CI of Hazard ratio**
SDMA (μg/dl)	14	0.0089^*^	3.52	1.460–8.476
sCr (μmol/L)	124	0.0211	2.675	0.999–7.160
sPhos (mmol/L)	1.5/1.5/1.6/1.9	0.0074^*^	3.25	1.306–8.076
UPC	0.5	0.7912	0.88	0.346–2.224
Hematocrit (L/L)	0.37	<0.0001^*^	8.51	1.094–66.19
SBP (mmHg)	140	0.9891	1.01	0.409–2.476
BCS (9/9)	4	0.0230^*^	2.68	1.075–6.684

*BCS, body condition score; SBP, systolic blood pressure; sCr, serum creatinine; SDMA, symmetric dimethylarginine; sPhos, serum phosphorus; UPC, urine protein: creatinine ratio*.

c *Intervals for serum phosphorus were in accordance to the CKD stage based on IRIS 2019 criteria*.

* *p < 0.05, statistically significant differences based on log-rank test*.

At inclusion time and during the course of the study, patients were staged and therapeutically managed according to the IRIS guidelines criteria proposed in 2016–2017 (5, 18), as these were in force at the time the study took place. The recent update on IRIS criteria in 2019 has certain differences with the previous criteria, allowing a comparison between them. Henceforth, it was observed that at *T*_0_ 86.1% (31/36) of the cases studied revealed a coincidence in CKD staging with the use of both criteria. However, when analyzing the coincidence in staging within each CKD stage, lower percentages of coincidence were observed, showing a greater discrepancy in stages II (50%) and III (25%) (Table 4). When analyzing the progression of the disease with the use of both criteria, 47.2% (17/36) of progression was evidenced with IRIS 2019 staging criteria, while 38.9% (14/36) was identified with the use of IRIS 2017 staging criteria, evidencing an 8.3% discrepancy between the current and past criteria.

**Table 4:** Frequency of cases classified in each CKD stage according with IRIS 2017 and 2019 staging criteria and the percentage of coincidence between the two classifications.

	**IRIS Staging criteria 2017**	**IRIS Staging criteria 2019**	**% Coincidence between criteria**
CKD stage I	22	20	90.9
CKD stage II	4	8	50
CKD stage III	4	3	75
CKD stage IV	6	5	83.3

## Discussion

Chronic kidney disease was progressive and tended to lead to death in advanced stages. However, its early detection enabled to achieve stability when RF were efficiently managed. Considering susceptibility RF, a median age of 10 years was observed in the studied population, likewise what other authors have reported (11, 15, 25). No sex or breed predisposition was observed (2, 26). The frequency of breeds identified was simply related to the preferences of owners of the area where this study was conducted. Small breeds were the most frequently observed ones, as other reports mention. However, an association between these factors has not yet been described (25, 27). Concurrent diseases identified as initiation RF matched with those reported by other authors, including periodontal disease, heart disease, neoplasms, pyometra, diabetes mellitus, and leptospirosis (2, 27, 28). The most frequently observed pathologies in the studied population were inflammatory/infectious diseases, evidencing the presence of two or more of these pathologic processes in 66.7% of the cases simultaneously.

The general and specific mortality observed in this study was lower than reports published by other authors (2, 15, 28). These may be related to the large proportion of pre-azotemic patients included in this study, opposed to other publications which analyzed only azotemic patients, that tend to be more likely to die (2, 11, 15). The high specific mortality noticed in patients in advanced CKD stages was related to the presence of end-stage RF, such as anemia, together with other organic alterations associated with uremic toxin accumulation (8, 29). Survival of patients who died secondary to CKD severity was similar to what was described by Fiocchi et al. (30). Nevertheless, longer survival times have been described in CKD patients (2). This discrepancy can be associated with the fact that in this study, specific mortality was only represented by patients in CKD stages III and IV, which tend to have a shorter survival related to the disease severity, according to what other authors have reported (2, 11, 15). In addition, survival time in advanced CKD stages (59–702 days range) matched with that previously described by Polzin (1).

When analyzing the behavior of interest variables among CKD stages at the time of diagnosis, it was noticed that GFR markers (SDMA, sCr, and serum urea) exhibited differences between early and advanced CKD stages, associated with the different degree of renal function decline present in each CKD stage and its relationship to various grades of uremic toxin accumulation (31). Concerning serum phosphorus concentration, a significant difference was noticed between patients in early CKD stages and CKD stage IV, evidencing its increment as CKD severity worsened. This was associated with the described dependence between phosphorus excretion capability and renal function, observing hyperphosphatemia's enhancement as GFR decreased (9). It should be noted that evidences of hyperphosphatemia were present in all CKD stages, mainly in advanced ones, in line with other literature (9, 32). Moreover, hyperphosphatemia was detected in cases included in stage I of CKD, which coincides with what was reported by Cortadellas et al. (12) and Foster (33) regarding the possibility of secondary renal hyperparathyroidism being present since early stages of CKD, developing before the onset of azotemia and even before hyperphosphatemia becomes evident. Therefore, according to Cortadellas et al. (12), starting with this RF's therapeutic management from early CKD stages should be considered, seeking to maintain serum phosphorus concentrations within the reference interval for each CKD stage, still more studies that fully evaluate changes in phosphate homeostasis are required (6).

Regarding USG, a significant difference was observed between cases in CKD stage I and advanced stages. Moreover, marked hypersthenuria (USG >1.030) was noticed in half of the cases classified in stage I (7/15), associated with the fact that in early CKD stages, the ability to concentrate urine can still be preserved (6, 21). Concerning the hematocrit, marked anemia was evidenced mainly in advanced CKD stages, in accordance with previous reports (15, 30). This finding was associated with the fact that increased CKD severity relates to a greater number of factors that favor the development of anemia. These factors involve erythropoietin production decrement due to the loss of functional renal parenchyma, erythrocyte half-life reduction secondary to uremia and hyperphosphatemia, blood losses secondary to uremic gastroenteritis, and iron deficiency associated with blood losses and malnutrition secondary to hyporexia or anorexia (8, 10), enhancing the aggravation of anemia. Furthermore, in advanced CKD stages, several patients presented low BCS and cachexia, related to the frequent presence of hyporexia or anorexia, gastroenteritis secondary to uremic toxins retention, dehydration, and biochemical abnormalities, such as metabolic acidosis, all of which favor BCS loss (8). The characteristic proinflammatory state present in CKD should be considered to play an important role in the development of hyporexia or anorexia and cachexia (34–36), considering likewise sarcopenia as a cause of decreased BCS in these patients (35).

### Risk Factors Involved in CKD Progression

Concurrent diseases considered CKD initiation RF identified in patients that showed progression, evidenced different pathophysiological mechanisms related to renal injury generation and damage progression (37–40). During early CKD stages, serum SDMA concentration was the only analyte which displayed differences among studied. The variation observed was associated with the disease progression evidenced in 35.7% (10/28) of the patients in these stages (31). Although the GFR markers used in this study have the same limitation to detect early changes in the decrease in GFR since they have a proportional relationship with it (6), SDMA seemed to be able to detect changes in GFR compatible with early renal function decline (41), and when assessing the evolution of its serum concentration over time. Identifying increases in this analyte could be a good indicator to start nephroprotective therapy as soon as possible, such as renal diet intervention (13).

Moreover, although a slight difference was evidenced in the hematocrit as time passed in early CKD stages, this finding was not considered clinically relevant. It should be mentioned that in these patients, the presence of concurrent diseases, such as advanced cardiopathies and/or chronic inflammatory diseases, could have affected erythropoiesis secondary to erythropoietin resistance caused by systemic chronic inflammation (34, 42). In advanced CKD stages, the only associated CKD RF that presented differences among study times was the hematocrit. Not only was the presence of anemia exhibited but also the worsening of this condition in more than half of these patients, i.e., 62.5% (5/8). It should be noted that despite carrying out therapeutic management to control anemia and factors involved in its development, such as hyperphosphatemia, uremic gastroenteritis, caloric-protein malnutrition, and systemic inflammatory conditions (43), such control was not achieved. Therefore, it can be inferred that during advanced CKD stages, therapeutic interventions concerning anemia management may prove to be inefficient.

### Risk Factors Associated With Survival in CKD

In this study, increased serum SDMA was associated with a decreased survival time, since the increase in its serum concentration correlates well with GFR decline (44), which in turn relates to increased death risk associated with CKD severity (2, 11, 15). Furthermore, SDMA is also a guanidine with uremic toxin potential and can generate cellular damage (45). It has been described that SDMA favors nitric oxide production decrement, stimulating reactive oxygen species production and a proinflammatory effect (46). The presence of hyperphosphatemia was also related to a decreased survival time, in line with what other authors have reported (15). This is attributable to the clinical complications that it entails, such as renal secondary hyperparathyroidism development and its consequences, i.e., renal osteodystrophy, tissue mineralization (33), and aggravation of anemia (10, 47). Furthermore, the presence of anemia was also associated with a decreased survival time (8, 48). This can be related to the harmful effects that arise from the decreased tissue oxygen supply and oxidative stress that occurs secondarily to anemia (8, 49). Moreover, low BCS was associated with decreased survival time, in line with what other authors have reported (11), and could be associated with the inflammatory, catabolic, and oxidative state that occurs secondarily to cachexic states (35), all related to a lower survival time (34).

### Comparison Between IRIS 2017 and 2019 Criteria for CKD Staging

A discrepancy in patients' distribution throughout the different CKD stages between IRIS criteria published in 2017 and 2019 (7, 20), mainly between CKD stages II and III, was noted. In addition, a slight increase in the disease progression (8.3%) was evidenced with the use of IRIS 2019 criteria, associated with the broadening of SDMA and sCr reference intervals, especially in stage II CKD cases. It is worth mentioning that the variation in the disease staging, generated by both criteria, did not involve relevant changes in therapeutic management prescribed for the patients studied.

In this study, CKD progression and advanced stages of the disease were found to be associated to increased severity of outcomes. The CKD diagnosis based on the persistent finding of abnormalities in disease early markers, such as SDMA and/or renal proteinuria, and timely therapeutic management of RF allowed for CKD stabilization, reducing progression to advanced stages, and favoring higher survival rates.

## Data Availability Statement

This study is part of the research project 4121/2016SF registered in the Secretary of Research and Advanced Studies of the Universidad Autónoma del Estado de México (UAEM). The datasets presented in this study can be found in the Institutional Repository-UAEM: http://ri.uaemex.mx/handle/20.500.11799/105581.

## Ethics Statement

Ethical review and approval was not required for the animal study because Medical management was carried out to diagnose CKD and concomitant pathologies of the studied animals, as well as to therapeutically manage factors involved in the aggravation of this disease, according to IRIS recommendations for that matter. No experimental interventions nor additional management generating pain or stress was performed to the studied cases, following the internationally established high standards of good practice and animal welfare, the Institutional Committee for the Care and Use of Laboratory Animals of the referred institution guidelines, and informed consent of owners was obtained. Written informed consent was obtained from the owners for the participation of their animals in this study.

## Author Contributions

JD-Á-C conceived the study. SP-P and AP-S collected the data. SP-P, IQ-H, and JD-Á-C analyzed the data and interpreted the results. SP-P and JD-Á-C wrote the first draft of the paper. AP-S, IQ-H, and SR-M revised the paper. All authors approved the final version of the manuscript for submission.

## Conflict of Interest

The authors declare that the research was conducted in the absence of any commercial or financial relationships that could be construed as a potential conflict of interest.

## Footnotes

^1^Renal therapeutic diet prescribed in the studied patients: Hill's™ Prescription Diet™ k/d™ canine (*n* = 13), Royal Canin Veterinary Diet® Renal Support™ canine (*n* = 18), and Purina Pro Plan Veterinary Diet NF Kidney Function Canine Formula (*n* = 5). During the course of the study some shifts among commercial brands were made because of patients' preferences and availability of the product.

^2^Ipakitine www.vetoquinolmx.com/content/ipakitine®-7090-001.

## Abbreviations

BCS: body condition score
G1, G2, G3, G4: groups 1, 2, 3 and 4
GFR: glomerular filtration rate
IRIS: International Renal Interest Society
RF: risk factor
SBP: systolic blood pressure
sCr: serum creatinine
SDMA: symmetric dimethyl arginine
*T*_0_: initial time
*T*_F_: final time
UPC: urine protein:creatinine ratio
USG: urinary specific gravity.

## References

[1] Polzin DJ. Chronic kidney disease in small animals. Vet Clin North Am Small Anim Pract. (2011) 41:15–30. 10.1016/j.cvsm.2010.09.004 21251509

[2] O'Neill D Elliott J Church D Mcgreevy P Thomson P Brodbelt D. Chronic kidney disease in dogs in UK veterinary practices: prevalence, risk factors, and survival. J Vet Intern Med. (2013) 27:814–21. 10.1111/jvim.12090 23647231

[3] Grauer GF. Early detection of renal damage and disease in dogs and cats. Vet Clin Small Anim. (2005) 35:581–96. 10.1016/j.cvsm.2004.12.013 15833560

[4] Syme H. International Renal Interest Society (IRIS). Early CKD Diagnosis. (2019). Available online at: http://www.iris-kidney.com/education/early_diagnosis.html (accessed April, 2021).

[5] Syme H. Iris-kidney.com. International Renal Interest Society (IRIS). Early CKD Diagnosis. (2016). Available online at: http://www.iris-kidney.com/education/early_diagnosis.html (accessed November, 2017).

[6] Sargent HJ Elliot J Jepson RE. The new age of renal biomarkers: does SDMA solve all of our problems? J Small Anim Pract. (2021) 62:71–81. 10.1111/jsap.13236 33184865

[7] International Renal Interest Society (IRIS). IRIS Staging of CKD (modified 2019). (2019). Available online at: http://www.iris-kidney.com/pdf/IRIS_Staging_of_CKD_modified_2019.pdf (accessed April, 2021).

[8] Bartges J. Chronic kidney disease in dogs and cats. Vet Clin North Am Small Anim Pract. (2012) 42:669–92. 10.1016/j.cvsm.2012.04.008 22720808

[9] Geddes R Finch N Syme H Elliott J. The role of phosphorus in the pathophysiology of chronic kidney disease. J Vet Emerg Crit Care. (2013) 23:122–33. 10.1111/vec.12032 23464730

[10] King L Giger U Diserens D Nagode L. Anemia of chronic renal failure in dogs. J Vet Intern Med. (1992) 6:264–70. 10.1111/j.1939-1676.1992.tb00350.x 1432900

[11] Parker V Freeman L. Association between body condition and survival in dogs with acquired chronic kidney disease. J Vet Intern Med. (2011) 25:1306–11. 10.1111/j.1939-1676.2011.00805.x 22092621

[12] Cortadellas O Fernández del Palacio M Talavera J Bayón A. Calcium and phosphorus homeostasis in dogs with spontaneous chronic kidney disease at different stages of severity. J Vet Intern Med. (2010) 24:73–9. 10.1111/j.1939-1676.2009.0415.x 19925576

[13] Hall J MacLeay J Yerramilli M Obare E Yerramilli M Schiefelbein H. Positive impact of nutritional interventions on serum symmetric dimethylarginine and creatinine concentrations in client-owned geriatric dogs. PLoS ONE. (2016) 11:e0153653. 10.1371/journal.pone.0153653 27088214 PMC4835100

[14] International Renal Interest Society (IRIS). Treatment Recommendations for CKD in Dogs. (2019). Available online at: http://www.iris-kidney.com/pdf/IRIS-DOG-Treatment_Recommendations_2019.pdf (accessed April, 2021).

[15] Rudinsky A Harjes L Byron J Chew D Toribio R Langston C. Factors associated with survival in dogs with chronic kidney disease. J Vet Intern Med. (2018) 32:1977–82. 10.1111/jvim.15322 30325060 PMC6271312

[16] Ryan S Bacon H Edenburg N Hazel S Jouppi R Lee N. WSAVA Animal Welfare Guidelines for companion animal practitioners and veterinary teams. J Small Anim Pract. (2019) 60:E1–46. 10.1111/jsap.12998 30859578

[17] Johnson CA Levey AS Coresh J Levin A Lau J Eknoyan G. Clinical practice guidelines for chronic kidney disease in adults: part I. Definition, disease stages, evaluation, treatment, and risk factors. Am Fam Physician. (2004) 70:869–876. 15368726

[18] Roura X. International Renal Interest Society (IRIS). IRIS CKD Risk Factors. (2019). Available online at: http://www.iris-kidney.com/education/risk_factors.html (accessed October, 2019).

[19] Acierno M Brown S Coleman A Jepson R Papich M Stepien R. ACVIM consensus statement: guidelines for the identification, evaluation, and management of systemic hypertension in dogs and cats. J Vet Intern Med. (2018) 32:1803–22. 10.1111/jvim.15331 30353952 PMC6271319

[20] Elliot J Watson ADJ. Chronic kidney disease: International renal interest society staging and management. In: Bonagura JD Twedt DC editor. Kirk's Current Veterinary Therapy XV. St. Louis, MO: Elsevier (2014). p. 857–63.

[21] Elliot J White J. International Renal Interest Society (IRIS). IRIS staging system - overview of the IRIS staging system for CKD (revised 2019). (2019). Available online at: http://www.iris-kidney.com/education/staging_system.html (accessed April, 2021).

[22] Lees G Brown S Elliott J Grauer G Vaden S. Assessment and management of proteinuria in dogs and cats: 2004 ACVIM forum consensus statement (small animal). J Vet Intern Med. (2005) 19:377–85. 10.1111/j.1939-1676.2005.tb02713.x 15954557

[23] Zatelli A Roura X D'lpoolito P Berlanda M Zini E. The effect of renal diet in association with enalapril or benazepril on proteinuria in dogs with proteinuric chronic kidney disease. Open Vet J. (2016) 6:121–7. 10.4314/ovj.v6i2.8 27540513 PMC4980477

[24] Lourenco BN Coleman AE Brown SA Schmiedt CW Parkanzky MC Creevy KE. Efficacy of telmisartan for the treatment of persistent renal proteinuria in dogs: a double-masked, randomized clinical trial). J Vet Intern Med. (2020) 36:2478–6. 10.1111/jvim.15958 33165969 PMC7694823

[25] Bartlett P Van Buren J Bartlett A Zhou C. Case-control study of risk factors associated with feline and canine chronic kidney disease. Vet Med Int. (2010) 2010:957570. 10.4061/2010/957570 20885927 PMC2946592

[26] Pelander L Ljungvall I Egenvall A Syme H Elliott J Häggström J. Incidence of and mortality from kidney disease in over 600,000 insured Swedish dogs. Vet Rec. (2015) 176:656. 10.1136/vr.103059 25940343

[27] Glickman L Glickman N Moore G Lund E Lantz G Pressler B. Association between chronic azotemic kidney disease and the severity of periodontal disease in dogs. Prev Vet Med. (2011) 99:193–200. 10.1016/j.prevetmed.2011.01.011 21345505

[28] Sosnar M Kohout P RuŽička M Vrbasová L. Retrospective study of renal failure in dogs and cats admitted to University of Veterinary and Pharmaceutical Sciences Brno during 1999-2001. Acta Vet Brno. (2003) 72:593–8. 10.2754/avb200372040593

[29] Finco D Brown S Brown C Crowell W Cooper T Barsanti J. Progression of chronic renal disease in the dog. J Vet Intern Med. (1999) 13:516–28. 10.1111/j.1939-1676.1999.tb02204.x 10587250

[30] Fiocchi E Cowgill L Brown D Markovich J Tucker S Labato M. The use of darbepoetin to stimulate erythropoiesis in the treatment of anemia of chronic kidney disease in dogs. J Vet Intern Med. (2017) 31:476–85. 10.1111/jvim.14681 28256075 PMC5354051

[31] Relford R Robertson J Clements C. Symmetric dimethylarginine: improving the diagnosis and staging of chronic kidney disease in small animals. Vet Clin North Am Small Anim Pract. (2016) 46:941–60. 10.1016/j.cvsm.2016.06.010 27499007

[32] Harjes L Parker V Dembek K Young GS Giovaninni LH Kogika MM. Fibroblast growth factor-23 concentration in dogs with chronic kidney disease. J Vet Intern Med. (2017) 31:784–90. 10.1111/jvim.14707 28419560 PMC5435078

[33] Foster JD. Update on mineral and bone disorders in chronic kidney disease. Vet Clin North Am Small Anim Pract. (2016) 46:1131–49. 10.1016/j.cvsm.2016.06.003 27436330

[34] Akchurin O Kaskel F. Update on inflammation in chronic kidney disease. Blood Purif. (2015) 39:84–92. 10.1159/000368940 25662331

[35] Morley J Thomas D Wilson M. Cachexia: pathophysiology and clinical relevance. Am J Clin Nutr. (2006) 83:735–43. 10.1093/ajcn/83.4.735 16600922

[36] Silverstein D. Inflammation in chronic kidney disease: role in the progression of renal and cardiovascular disease. Pediatr Nephrol. (2009). 24:1445–52. 10.1007/s00467-008-1046-0 19083024

[37] Pouchelon J Atkins C Bussadori C Oyama MA Vaden SL Bonagura JD. Cardiovascular-renal axis disorders in the domestic dog and cat: a veterinary consensus statement. J Small Anim Pract. (2015) 56:537–52. 10.1111/jsap.12387 26331869 PMC4584495

[38] Prudic R Saba C Lourenço B Bugbee A. Prevalence of proteinuria in a canine oncology population. J Small Anim Pract. (2018) 59:496–500. 10.1111/jsap.12840 29608792

[39] Sant'Anna R Vieira A Oliveira J Lilenbaum W. Asymptomatic leptospiral infection is associated with canine chronic kidney disease. Comp Immunol Microbiol Infect Dis. (2019) 62:64–7. 10.1016/j.cimid.2018.11.009 30711048

[40] Yang C Wu M Pan M. Leptospirosis renal disease. Nephrol Dial Transplant. (2001) 16(Suppl. 5):73–7. 10.1093/ndt/16.suppl_5.73 11509689

[41] Hall JA Yerramilli M Obare E Yerramilli M Almes K Jewell DE. Serum concentrations of symmetric dimethylarginine and creatinine in dogs with naturally occurring chronic kidney disease. J Vet Intern Med. (2016) 30:794–802. 10.1111/jvim.13942 27103204 PMC4913574

[42] Rosner M Ronco C Okusa M. The role of inflammation in the cardio-renal syndrome: a focus on cytokines and inflammatory mediators. Semin Nephrol. (2012) 31:70–8. 10.1016/j.semnephrol.2011.11.010 22365165

[43] Quimby JM. Update on medical management of clinical manifestations of chronic kidney disease. Vet Clin North Am Small Anim Pract. (2016) 46:1163–81. 10.1016/j.cvsm.2016.06.004 27593576

[44] Hokamp J Nabity M. Renal biomarkers in domestic species. Vet Clin Pathol. (2016) 45:28–56. 10.1111/vcp.12333 26918420

[45] Schepers E Speer T Bode-Böger S Fliser D Kielstein J. Dimethylarginines ADMA and SDMA: the real water-soluble small toxins? Semin Nephrol. (2014) 34:97–105. 10.1016/j.semnephrol.2014.02.003 24780466

[46] Kielstein J Fliser D Veldink H. Asymmetric dimethylarginine and symmetric dimethylarginine: axis of evil or useful alliance? Semin Dial. (2009) 22:346–50. 10.1111/j.1525-139X.2009.00578.x 19708979

[47] Chalhoub S Langston C Eatroff A. Anemia of renal disease. What it is, what to do and what's new. J Feline Med Surg. (2011) 13:629–40. 10.1016/j.jfms.2011.07.016 21872790 PMC10832667

[48] Sato Y Fujimoto S Konta T Iseki K Moriyama T Yamagata K. Anemia as a risk factor for all-cause mortality: Obscure synergic effect of chronic kidney disease. Clin Exp Nephrol. (2018) 22:388–94. 10.1007/s10157-017-1468-8 28815319

[49] Patel T Singh A. Anemia in chronic kidney disease: new advances. Heart Failure Clin. (2010) 6:347–57. 10.1016/j.hfc.2010.02.001 20630409

